# SEARCHPATTOOL: a new method for mining the most specific frequent patterns for binding sites with application to prokaryotic DNA sequences

**DOI:** 10.1186/1471-2105-8-354

**Published:** 2007-09-20

**Authors:** Fathi Elloumi, Martha Nason

**Affiliations:** 1 Research Technology Branch, National Institute of Allergy and Infectious Diseases, National Institutes of Health, 9000 Rockville Pike, Blg50/R5505, Bethesda, MD 20892, USA; 2 Biostatistics Research Branch, Office of Clinical Research, National Institute of Allergy and Infectious Diseases, National Institutes of Health, 6700B Rockledge Dr. MSC 7609, Bethesda, MD 20892-7609, USA

## Abstract

**Background:**

Computational methods to predict transcription factor binding sites (TFBS) based on exhaustive algorithms are guaranteed to find the best patterns but are often limited to short ones or impose some constraints on the pattern type. Many patterns for binding sites in prokaryotic species are not well characterized but are known to be large, between 16–30 base pairs (bp) and contain at least 2 conserved bases. The length of prokaryotic species promoters (about 400 bp) and our interest in studying a small set of genes that could be a cluster of co-regulated genes from microarray experiments led to the development of a new exhaustive algorithm targeting these large patterns.

**Results:**

We present Searchpattool, a new method to search for and select the most specific (conservative) frequent patterns. This method does not impose restrictions on the density or the structure of the pattern. The best patterns (motifs) are selected using several statistics, including a new application of a z-score based on the number of matching sequences. We compared Searchpattool against other well known algorithms on a *Bacillus subtilis *group of 14 input sequences and found that in our experiments Searchpattool always performed the best based on performance scores.

**Conclusion:**

Searchpattool is a new method for pattern discovery relative to transcription factor binding sites for species or genes with short promoters. It outputs the most specific significant patterns and helps the biologist to choose the best candidates.

## Background

The availability of complete genomic sequences has opened the door for computational methods to predict binding sites and understand gene regulation. Pattern-finding algorithms can be divided into two groups [1]: local multiple sequence alignment algorithms and exhaustive algorithms. Alignment based algorithms (e.g. Gibbs sampling, expectation maximization) may converge to a local maximum without always finding the best patterns [2,3]. Exhaustive algorithms are guaranteed to find the best patterns within certain constraints. Brazma formalized the problem of pattern discovery as a classification one [4]. Rigoutsos defined the problem as follows [5]: Given a database D (set of sequences), a set of events E (four nucleotides for DNA sequences) and an evaluation function F (that measures the degree of similarity between two events) the task is to determine interesting patterns of events which are contained in D. An interesting pattern is, for example, a frequent and statistically significant pattern. A frequent pattern is one that appears in a minimum number of records (sequences). This minimum number is called the threshold, or minimum support. A significant pattern is one that occurs too frequently to be attributed to chance alone, as judged by having a high statistical score. Frequent patterns can be classified in three categories [6]: all frequent patterns, the closed frequent patterns where all extensions have smaller support, and finally the maximal frequent patterns that are not contained in other patterns. Regular expressions are sometimes used to define the patterns. The pattern can contain the events of E (the fixed alphabet) and ambiguous characters (N, R, W...). The character N (or '.') is a wild-character that can represent any event. The density of a pattern is its number of non-wild-characters.

Most exhaustive algorithms operate by enumerating the solution space. For example, after fixing a minimum support they search for a simple frequent pattern (singleton), extend it, and check if the extended pattern is frequent. The process is then repeated. As the lengths of the patterns increase the running time grows. Some programs use pruning techniques or impose constraints on the kind of patterns so the performance is improved. Well known exhaustive algorithms include Ymf, Weeder and Mitra [7-9]. Others, like Pratt, Teiresias and Splash are not dedicated to patterns relative to binding sites [10-12]. Some programs are based on mining sequential patterns [13], like the Wang program [14], or Tomms that uses a top-down pattern enumeration [6].

In this work we are looking for TFBS relative to prokaryotic species. A summary of the motifs in TF databases (such as RegulonDB and DBTBS) reveal that approximately 67% of the motifs are at least 15 bp [15,16]. Based on the observations found in these databases and relevant papers, it seems that the motifs for prokaryotic species are likely to be large and contain a number of conserved bases. Based on a search in DBTBS and RegulonDB, we find that 77% of the motifs have at least 2 conserved bases for *Bacillus subtilis*. For monad patterns without flexible gaps, the corresponding percentage is 88%. 39% of the motifs begin and end with a conserved base. For *E. coli*, we find that 54% of the motifs have at least 2 conserved bases and 33% of the motifs begin and end with a conserved base.

In general the motifs do not follow a specific type. In DBTBS we find that about 29% of the motifs have few spacers (between one and seven) in the middle and 21% of the motifs have flexible gaps. Approximately 19% of motifs are palindromic.

Local multiple algorithms may be suitable for our application; however, they are not guaranteed to find optimal solutions. Currently available exhaustive algorithms impose some constraints in order to limit the search time and to have good performance: these did not fit our requirements because they are not capable of finding long motifs, as our context demands. For example, in Ymf a target pattern or motif is a string of length 6–8 over the alphabet {A,C,G,T,R,Y,S,W} with 0 to 11 character 'N's inserted in the center and a limited number of R, Y, S, W characters (Ex: CGGNNNNNNSCG). Weeder enumerates all patterns up to a maximum length (max 12) with a fixed number of substitutions (max 4) of the sites when compared to the motif. Mitra searches for contiguous strings (monads) with a fixed number of mismatches (substitutions) and a minimum value of occurrences. On synthetic and biological data Mitra succeeded in retrieving a monad of size 18 bp; however, when the motif is larger and the number of mismatches is higher it consumes more resources and may fail to retrieve the motif. This algorithm can also find dyads or composite patterns where a group of monad patterns occur near each other with spacers in the middle.

None of the available algorithms suited the demands of our context exactly. As we could not find an existing exhaustive search capable of finding long motifs, and knowing that we are interested in searching patterns for binding sites relative to a small set of genes (around 100 genes) believed to be co-regulated or share a common pathway or a biologic function, with short promoter regions (between 400–1000 bp), we decided to develop a new exhaustive algorithm that looks for short and large motifs with at least 2 conserved bases. Our algorithm does not require that the user specify the exact length and structure of the motifs, except that they must begin and end with conserved bases. The new algorithm needs to be run just one time and will search for the most conservative (specific) motifs (with conserved bases) that are common to a minimum number of the input sequences.

The length of those patterns found by this new algorithm may vary from 2 to 30 bp or more. The format of the pattern is E (E ∪ '.')* E where E represent an event from the exact alphabet, the character '.' is the wild-character N, ∪ represents the union operator and * the repetition from 0 to n times. We assume that a pattern must begin and end with a conserved nucleotide. About 40% of the patterns for *Bacillus subtilis *fit strictly within this heuristic. While not every possible pattern fits this heuristic, our algorithm is intended to look for the most specific or conservative large frequent patterns that does. Searchpattool will retrieve known and unknown motifs that fit this format. If a desired motif contains at least 2 conserved bases that are not located at the first and last positions, Searchpattool is able to discover the core region that contained the conserved bases. In the case where the motif does not contains conserved bases at all, our algorithm will retrieve the most specific patterns that are shared by a smaller number of sequences. By specifying a minimum support value less than 100%, Searchpattool will output the most specific pattern with different support values.

The algorithm consists of 4 main steps: first, it finds all frequent patterns of different sizes that contain exactly 2 conserved nucleotides; second, it makes them more specific by replacing the wild-characters with conserved nucleotides; third, it scores all frequent patterns based on summary statistics, and fourth it outputs the best user-specified number of patterns ordered by their statistical score. We follow the same statistical method (z-score) used by Van Helden to score the total number of occurrences for a motif and we introduce a new application of the z-score by applying it to each motif's support [17]. The support statistic is very interesting because it is based on the matching number of sequences.

The search for frequent patterns is done in the positive and negative strands of the input sequences that can be of different sizes. The algorithm provides, for each pattern, useful information including length, support, density, total number of occurrences, positions, z-score for the support and z-score for the total number of occurrences, sites, profiles (matrix of frequency), mean of information content and consensus. The algorithm compares the best patterns and provides information about their similarity and reverse complements. In this work we consider that a pattern and its reverse complement are different candidates and are scored independently.

The format of the pattern is similar to that used by Teiresias [11]; however, Searchpattool and Teiresias are different. Teiresias outputs all maximal (or closed) <L,W> frequent patterns where each sub-pattern with length W contains at least L residues or conserved bases. The user has to specify L, W and the value of minimum support.

In addition, we developed an associated tool to compute the p-value associated with the z-score of the support for the best motifs by comparing to a similar search on randomly generated data. We can compute a p-value for each ranked score by comparing it to a null distribution of similarly ranked scores from simulations. This additional step provides an additional safe-guard against the risk of erroneously identifying patterns as common due only to the large number of patterns being considered, and is an improvement over other existing algorithms. Based on the p-value of the z-score and the previous information about the pattern including its support value and its similarity with other patterns, the user can make inferences about the strength of evidence that their best patterns are truly more common than chance would suggest, and can choose the best candidate or candidates for further biologic experiments.

We assessed Searchpattool by comparing its accuracy to three widely used local multiple algorithms (Meme, Motif Sampler, Consensus) [3,18,19]; and one exhaustive algorithm(Mitra) [9]. Meme uses an expectation-maximization algorithm, Motif sampler is a variant of a Gibbs sampler algorithm and Consensus is a greedy algorithm. Exhaustive algorithms allow searching for monad or composite motifs. Our algorithm should be compared to those that look for monad motifs. Most of these algorithms limit the size of monad motifs to 12 bp. Mitra can retrieve large monad patterns. Due to these limitations we chose to compare our algorithm to only Mitra.

We also studied Searchpattool's runtime and its number of patterns by varying different parameters including the minimum support, the maximum length of the pattern and the number of sequences.

## Results

### Algorithm

In order to run Searchpattool, the user has to specify the input sequences, the minimum support, the maximum length of patterns, the background probabilities for the sequence's species and finally (optionally) the number of patterns to output. Searchpattool has four steps:

#### Step 1: Search all E ('.')* E frequent patterns

**Figure 1:**
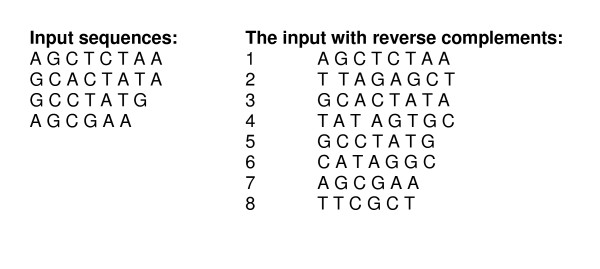
Example of an input sequences.

The algorithm searches all frequent patterns with only 2 nucleotides: one at the beginning and the second at the end with all possible numbers of wild-characters in the middle. The number of wild-characters is limited only by the user-specified maximum length (maxlen). There are 16*(maxlen-1) candidate patterns with 4 families of patterns: those that begin with A, C, G and T. Figure 1 shows an example of 4 input sequences, where we ran Searchpattool with a minimum support value of 2 and a maximum length value of 8. Searchpattool began by adding the reverse complement of the input sequences (see Figure 1), then searched all frequent patterns with only 2 conserved nucleotides. It enumerated all 4 families of patterns: those that begin with A, C, G and T. We show in Table 1 the results for a family of patterns that start with A.

**Table 1:** All frequent patterns for family A

**Pattern**	**Matches(No seq, 1st position)**	**support**	**total**
AA	[1,7] [7,5]	2	2
AG	[1,1] [2,3] [2,5] [4,4] [6,4] [7,1]	4	6
AT	[3,6] [4,2] [5,5] [6,2]	2	4
A.A	[2,3] [3,6] [4,2] [6,2]	3	4
A.C	[1,1] [2,5] [7,1]	2	3
A..G	[2,3] [4,2] [4,4] [6,2] [7,1]	4	5
A...C	[1,1] [2,3] [4,4]	2	3
A....A	[3,3] [7,1]	2	2

For each pattern, the occurrences of the patterns in the input sequences (Matches) -within the bracket you find the number of sequence (No seq) and the first position of the pattern (1st position)-, the number of matching sequences (support) and the total number of occurrences (total) are shown. All these patterns have a support greater or equal to 2.

#### Step 2: Deduce from all E ('.')* E frequent patterns the most specific ones

For each family we combine all patterns to have the most specific ones. We combine, if possible, each pattern P with all shorter patterns Q. P can be combined (or matched) to Q if the contents C_i_(Q) for all positions i for shorter pattern Q can be matched to those C_i _(P) in pattern P. C_i _(Q) matches C_i _(P) if they are the same or one of them is the wild-character '.'. For instance pattern "A.T.A" can be combined with pattern "AC" but not with pattern "A.C". When C_i _(Q) matches a C_i _(P) we can put at position i a more specific content by replacing the wild-character with a nucleotide. From "A.T.A" and "AC" we can have pattern "ACT.A". The idea is that if pattern P is combined with pattern Q then we can make a new pattern R that is more specific than P but not necessarily as frequent. The list of matches (positions) of R is the intersection of the matches of P and Q. We have to check if this list satisfies the minimum support. After that we distinguish 4 cases: First, if P and Q have the same list of positions then P is replaced by R and Q is deleted. Second, if the list of positions of R is the same as P then we replace P with R but we keep pattern Q. Third, if the list of positions of R is the same as Q then we add R as a new pattern, keep P and delete Q. Otherwise the new pattern R is added and P and Q are kept. New patterns are checked against existing ones to avoid redundancy. In our example (see Table 1), the pattern A.A can be combined with AA, AG and AT but the list of positions is null for AAA and contains only one position for AGA. This results only in a new pattern ATA that is more specific than A.A. Pattern A.C when combined with AG is updated to AGC since it keeps its positions. The most specific patterns for family A are shown in Table 2.

**Table 2:** All most specific frequent patterns for family A

**Pattern**	**Matches(No seq,1st position)**	**support**	**Total**
AA	[1,7] [7,5]	2	2
AG	[1,1] [2,3] [2,5] [4,4] [6,4] [7,1]	4	6
AT	[3,6] [4,2] [5,5] [6,2]	2	4
A.A	[2,3] [3,6] [4,2] [6,2]	3	4
**ATA**	[3,6] [4,2] [6,2]	2	3
**AGC**	[1,1] [2,5] [7,1]	2	3
A..G	[2,3] [4,2] [4,4] [6,2] [7,1]	4	5
**AG.G**	[2,3] [4,4] [7,1]	3	3
**ATAG**	[4,2] [6,2]	2	2
**A.AG**	[2,3] [4,2] [6,2]	3	3
**AG..C**	[1,1] [2,3] [4,4]	2	3
**AG.GC**	[2,3] [4,4]	2	2
A....A	[3,3] [7,1]	2	2

Bold patterns are the results of step 2.

For each family we assure that each pattern is made the most specific for its total number of occurrences but it can lead to a new more specific pattern with fewer occurrences or matching sequences. In our example, the pattern "A...C" becomes "AG..C" (with the same number of occurrences which is 3) and leads to a new pattern "AG.GC" (with only 2 occurrences).

#### Step 3: Scoring all frequent patterns

Given a set V of m sequences, a subset C (C ⊆ V) of size n, and a pattern P that occurs in 's' sequences from C and matches 'o' positions in C (including double strands), we can compute the probability of pattern P matching s or more sequences of C and the probability of P matching o or more positions in C. For each pattern we compute a z-score of the number of matching sequences -or support- (zs-sup) and a z-score of the total number of matching positions (zs-tot).

One issue with the latter score is the overlapping words. Pevzner defines an auto-correlation coefficient [20]. The presence of the degenerate symbol '.' increases the number of overlapping patterns. To avoid this problem we follow the formula of Van Helden and we count the number of occurrences of a motif while disregarding the overlapping positions [17]. For the former score we introduce a new formula (see Methods section).

#### Step 4: Output the patterns with best z-scores

The patterns can be ordered according to their z-score of the support (zs-sup) or the z-score of the total number of occurrences (zs-tot). In this paper, since our experiments are done in prokaryotic species and many transcriptions factor binding sites (TFBS) are rare we use the zs-sup score as our ordering criterion. The patterns are ordered according to the Z-score of the support (zs-sup) and the best user-specified numbers of patterns (default value 40 patterns) are selected. For each selected pattern we extract its sites, compute its matrix of frequencies, derive its consensus following the rules adapted from Cavener and calculate its information content score [21]. Since we have patterns of different lengths we take the mean (average) of the information content (MIC). Finally the selected patterns are compared to detect the reverse complements and measure the degree of their similarity. For each pattern P we check if it covers or extends (overlap 100%) another pattern Q and measure their degree of similarity by computing the average site similarity score. It is the same score function defined by Burset and Guigo and used to assess the performance quality of a motif finding algorithm (see average site performance in Methods section) [22,23]. A similarity score of 1 means that P covers totally Q. So P is an extension of Q. A zero score means that P does not cover Q at all. A score between 0 and 1 means that some sites of P cover some of Q. This scoring function is not symmetric.

A formal description of the algorithm is described in the Methods section.

### Predicting the best pattern

We compute the probabilities that the higher z-scores (zs-sup) can be reached by chance by the selection process. This is important because the motif selection process of our algorithm means that we cannot expect the distribution of the (for example) max(zs-sup) to have a standard normal distribution under the null hypothesis that there is no association between the sequences, so we cannot rely on the magnitude of the zs-sup from the chosen motifs to judge statistical significance (see methods section).

We suggest the p-value score be used as a first criterion to select the candidates for the best patterns. If two patterns have the same p-value one can choose the pattern with the highest support. Finally the user should check if the patterns are reverse complement and examine the similarity values between the patterns.

## Implementation

We developed Searchpattool and other programs with Borland C++ under Windows XP. We run it on a PC Pentium 4 (3,2 GHz). The main programs are:

SEARCHPATTOOL: outputs the most specific E (E U '.')* E formatted patterns ordered by their z-score of the support. We chose to limit the search to patterns of a maximum length of 40 bp. An input sequence should be in Fasta format (now up to 500 bp). The results are written as text files but can read by MS Excel or many other programs. The main files are:

- bestzssup.txt: contains the list of patterns with their length, density, support, z-scores and list of occurrences.

- Sites-positions.txt: contains the sites and the exact positions of the patterns

- Sites-for-logos.txt: contains just the sites in order to display logos

- Patt-profiles.txt: contains the frequency matrices of the patterns

- Patt-cons-mic.txt: contains the list of patterns plus their consensus and their mean information content

- Patt-rev-com.txt: indicates for each pattern its reverse complement on the list of patterns

- Patt-similarity.txt: measures the degree of similarity between the listed patterns

SEARCH_BEST_RANDOM_SCORES: a program that computes the best z-scores of the support (zs-sup) based on 1000 random samples. In order to compute the p-values of the z-score of the support we wrote an R script that generates random samples according to the background probabilities of the species and the specific lengths of the input sequences. The user can run this script and then call this program with the same parameters used in the Searchpattool call including the number of outputs.

COMPUTE_PVALUE: a program that computes the p-value of the best patterns (as judged by zs-sup) relative to the current patterns on our list by using the scores from the random data.

SELECT_BEST_ZS_SUP: a program that outputs the n best patterns ordered by their z-score of the support. A run of Searchpattool should precede it. This program is useful if the user wants to specify a number of outputs different from that used in the call of Searchpattool. It avoids searching again for all patterns with the same parameters.

SELECT_BEST_ZS_TOT: a program that outputs the best patterns ordered by their z-score of the total number of occurrences. A run of Searchpattool should precede it

**Figure 2:**
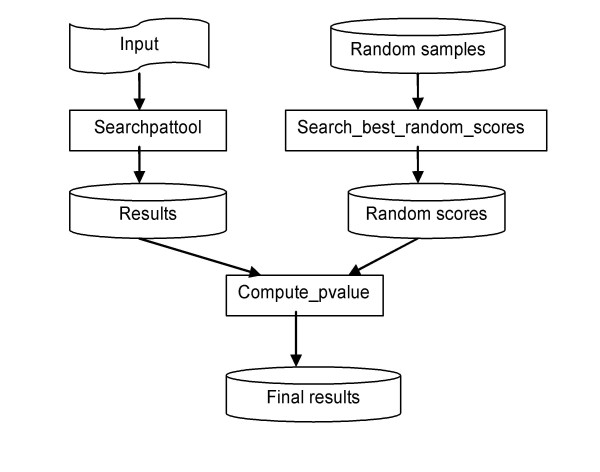
Overview of the main programs.

Figure 2 gives an overview of the order of use of the main programs.

## Testing

### Data experiments

DBTBS is a database of transcriptional regulation in *Bacillus subtilis *that provides information about well-known TFBS in this species [16]. In DBTBS, for each transcription factor we know the genes (or operons) that it binds on. After we selected 14 TF, we extracted upstream regions located at -400,-1 from the first codon and sometimes we extracted regions located at -400,+50 since some repressors are located downstream. We avoided overlap with upstream genes. We used Regulatory Sequence Analysis Tools (**RSAT**) to extract and purge the upstream of selected genes [24]. We checked the patterns sites cited in DBTBS and only used those that we found in the upstream. We present our input sequences in Table 3.

**Table 3:** Presentation of experiment datasets

**TF**	**Pattern Length**	**No genes (sequences)**	**No known Sites**	**No checked sites**	**Min-Len Sequence**	**Max-Len Sequence**	**Avr-Len Sequence**
SigL	17	6	6	6	124	240	197.6
comA	15	4	5	4	144	400	276.5
Hrca	27	2	2	2	119	238	178.5
zur	14	3	3	3	106	359	234.3
mntr	19	2	4	2	280	330	305
gltr	15	2	4	4	197	384	290.5
glnr	17	3	6	3	110	400	229.66
Spo0A	7	10	23	21	84	451	284.3
RocR	15	2	7	4	277	291	284
Fnr	16	5	6	6	186	246	211.6
CodY	11	4	4	4	179	451	356
Fur	20	20	23	21	82	451	231.65
DegU	20	14	15	11	133	451	335.85
TnrA	17	21	25	20	161	451	287.47

More details about the input sequences and the known checked sites are given in the additional files [see Additional file 1]. We designed two tests to compare the accuracy of Searchpattool to that of well known algorithms on these 14 input sequences.

### Test1: search for fixed length patterns

In the first test we compare Searchpattool to Meme, Motifsampler, Consensus and Mitra. We provide all algorithms with the exact known length of the target patterns. They share other common parameters including the input sequences, the search in the double strands and a report size of the best 40 patterns. All the algorithms were run using a background model of order 0 for *Bacillus subtilis *except Mitra that computes its own background model. Meme and Consensus are run twice varying each time the number of sites per sequence. Motif sampler is set to run 20 times and report each time the best 10 motifs. Mitra is set to run 5 times (varying the number of mismatches from 0 to 4) and reports each time the best 40 motifs. Searchpattool is run only one time. Since we are interested in searching for patterns for TFBS that are common to a set of genes believed to be a cluster of co-regulated genes from microarray experiments, specifying a minimum support value equal to 100% may not be appropriate for this example. We chose to specify a minimum support value less than 100% for two reasons. First, the input sequences may not represent the complete promoters or may not contain the motif due to imprecision in experiments or in the clustering process. Second, we are interested in looking for the most conservative motifs which are not necessarily shared by all the sequences. In our experiments we set the minimum support value to 60%, because it seemed to be a reasonable minimum level within a cluster for our context.

Since Searchpattool outputs patterns of different lengths we selected only 40 from those of the specified length. We then computed the p-value of their support z-scores and ordered them by p-value (increasing first criterion) and support (decreasing second criterion). Table 4 below summarizes the algorithm's parameters.

**Table 4:** Test1 setting parameters

	**Meme**	**Consensus**	**Motif sampler**	**Mitra**	**Searchpattool**
Run times	2	2	20	5	1
Number of site per sequence	zoops anr	0-n 1-n	Max n sites	no	no
Minimum number of occurrences	no	no	no	2–3	no
Minimum support	no	no	no	no	60%
Number of mismatches	no	no	no	0–4	no
Pattern length input	L*	L	L	L	Max L
Number of outputs	40	40	10 (x 20) Limited to 40	Best 40 patterns	Limited to 40 best zs-sup Ordered by p-value + and support -
Pattern length output	L	L	L	L	2 to L Limited to L

* L = exact known pattern length

Different statistics have been suggested to assess the performance quality of a motif finding algorithm [23]. Since all the patterns have the same length, we chose to follow Pevzner and used their nucleotide level performance score -ps- (see Methods section) [25]. The score will be 1 if the program finds the known sites whereas it will be 0 if it fails to retrieve any known sites. For each algorithm we record only the best performance score.

#### Searchpattool results

**Figure 3:**
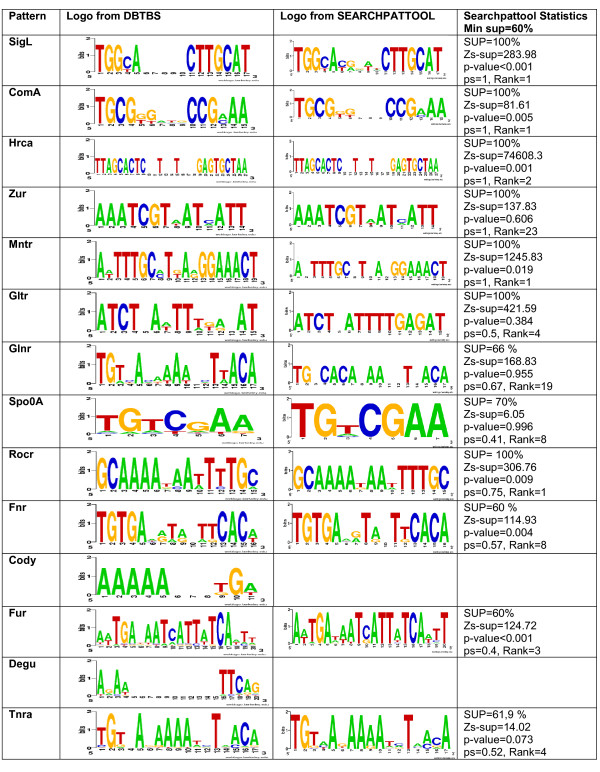
**Test1 Searchpattool results**. For each TF pattern, we provide its logo from DBTBS, its logo from Searchpattool, some statistics including the values of the support (SUP), z-score of the support (zs-sup), p-value, performance score (ps) and rank.

When the known motif is of the structure assumed by Searchpattool, Searchpattool succeeded in retrieving all its known sites. This is the case for 5 TF which are SigL, Coma, HrcA, Zur and Mntr. The patterns for Gltr and Glnr also follow Searchpattool's model, however, Searchpattool reports only a few known sites for them. The correct (or known) patterns for Gltr and Glnr are retrieved but are not selected because their z-scores are not among the best forty. For Gltr, the selected pattern has a performance score of 0.5, has a zs-sup value of 421.59 and is ranked 4. The correct pattern has a performance score of 1, has a zs-sup value of 14.51 and is ranked 819. The selected pattern is more specific than the correct one (see Gltr patterns in Figure 3).

The remaining seven TF known pattern don't follow Searchpattool's model, and as expected the algorithm cannot report all their known sites. However, Searchpattool succeeded in retrieving the most specific patterns that are similar to the correct ones for five; the exceptions are TF Cody and Degu, where it reported no results among the best 40 scored patterns. Figure 3 shows the main results for Searchpattool.

The z-score of the support (zs-sup) is a useful criterion to maximize in our experiments. However, we note that for patterns with many sites per sequence the z-score of the total number of occurrences (zs-tot) is higher than the z-score of the support (zs-sup) and that might affect the rank of the pattern. For example, when ordering the patterns by using the z-score of the total number of occurrences the rank of Gltr pattern becomes 225 (previously ranked 819 for the z-score of the support). Detailed results for Searchpattool test1 can be found in the additional files [see Additional file 2].

#### Comparison with other algorithms

**Figure 4:**
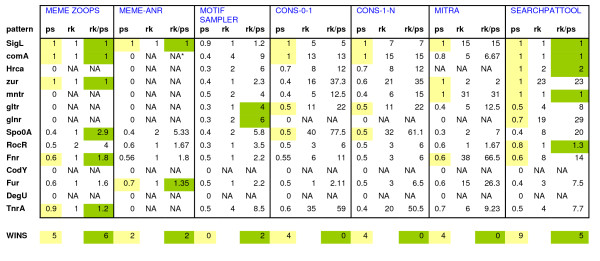
**Performance comparison of different programs for the search of 14 *Bacillus subtilis *patterns**. For each TF pattern, we indicate its performance scores (ps), rank (rk) and ratio rk/ps for respectively Meme (zoops and anr), Motif sampler (for 20 run), Consensus (o-1 and 1-n), Mitra (for 5 run) and Searchpattool. Yellow and green background colours indicate respectively the best performance score and the best ratio. *Na = not available

We ran the same fourteen input sequences on Meme, Motifsampler, Consensus and Mitra. Like Sinha and Tompa [26], for each pattern we declare a program the "winner" if it has the highest performance score. In order to take into account the rank of the pattern we compute the ratio of rank to performance score. We report the results for one run of each program, except for Motif Sampler (20 runs) and Mitra (5 runs). Figure 4 shows the results for the best performance scores for each program. When comparing the performance score, Searchpattool wins nine times, Meme (zoops) wins five times, Consensus wins four times, Mitra wins four times and Motif sampler does not win at all. Searchpattool outperforms all the other programs on three TF (hrca, glnr and rocr). In addition it identified the exact sites for five TF (with score of 1) more than Meme (three TF), Mitra (three TF) and Consensus (two TF). All algorithms fail to report results for Cody and Degu. When comparing the ratio rank/performance-score (rk/ps), Meme (zoops) is the winner, with six best ratios and the best ranking. Searchpattool follows next with five best ratios. For our datasets the performance of Meme zoops is better than that of Meme anr. We note that Mitra failed to retrieve Hrca the pattern because it is limited to a maximum length of 20 bp.

### Test2: search for maximum length patterns

A good tool should predict the correct patterns without requiring the user to specify their exact lengths. We tested the accuracy of Searchpattool against Meme when the correct length is not specified. We provided for each algorithm a maximum length for each searched pattern that is equal to the correct length plus 20%. Consensus, Motif Sampler and Mitra were not run because they don't allow for such a parameter. Meme was run twice (zoops and anr) and was set to output its best 40 patterns ordered by e-value. Searchpattool was set to select the 1000 best patterns based on z-score for support. These patterns were ordered by p-value (increasing first criterion) and support value (decreasing second criterion), and the best 40 ranked patterns were chosen. Table 5 summarizes the algorithms' parameters. In this test, since the correct length is not specified, the performance quality of the algorithms can be assessed at the site level. A predicted site overlaps a known site if they overlap by at least a defined percentage of the known site length. This percentage will take respectively the value of 100%, 75%, 50% and 25%. We used the average site performance which is the average between two scores: the sensitivity score and the predictive value scores (see Methods section) to assess the algorithms' performance in this test [22,23].

**Table 5:** Test2 setting parameters

	**Meme**	**Searchpattool**
Run times	2	1
Number of site per sequence	zoops anr	No
Minimum support	no	60%
Pattern length input	Maximum T*	Maximum T
Number of outputs	40	1000 best zs-sup Ordered by p-value + and support - Limited to 40

*T = L + 20%L

#### Searchpattool results

We checked if Searchpattool retrieved the known sites (with minimum overlap of 100%). We found that it succeeded in retrieving all known sites for four TF which are SigL, coma, HrcA and Mntr. It only retrieved some known sites for the rest of the patterns. The correct sites for Zur and Rocr TF are found but their p-value rank is greater than 40. The correct length is reported for five TF which are SigL, Glnr, Spo0A, Fur and Tnra. In fact if the known sites of a pattern have some common extensions to the right or left, Searchpattool will report first the larger patterns that are also the most specific with better z-scores. For instance, the predicted pattern for ComA TF has a length of 18 bp and is ranked 8, however, the pattern ranked 21 has the correct length which is 15. For ComA TF, Pattern 8 is an extension of pattern 21 with the same number of occurrences, has the best p-value and so has been selected. We remark also that Searchpattool reports pattern candidates for all TF including Cody and Degu.

**Figure 5:**
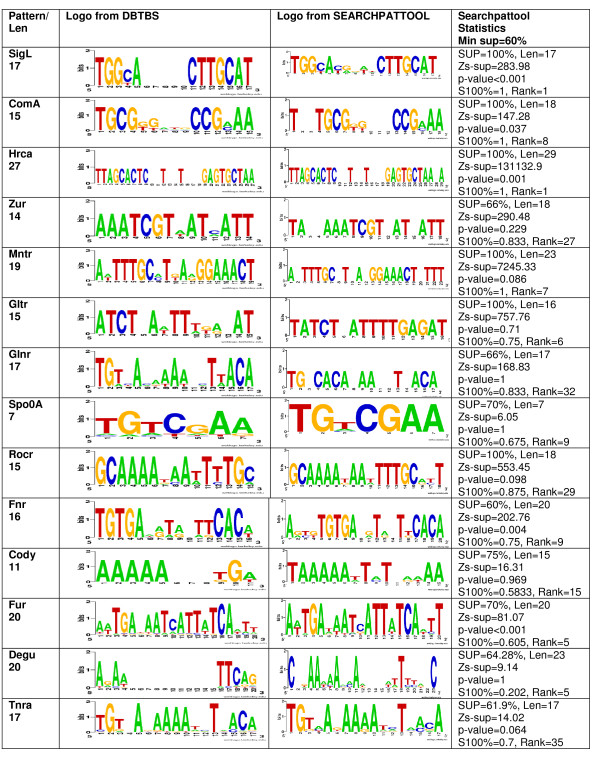
**Test2 Searchpattool results**. For each TF pattern, we provide its logo from DBTBS, its logo from Searchpattool, some statistics including the values of the support (SUP), length (len), z-score of the support (zs-sup), p-value, average site score for a minimum overlap of 100% (S100%) and rank.

Figure 5 shows the main results for Searchpattool. Detailed results for Searchpattool test2 can be found in the additional files [see Additional file 3].

#### Comparison with other algorithms

We run the same 14 input sequences on Meme (zoops and anr). We compared the performance of Searchpattool and Meme based on the average site performance score and by varying the percentage of overlap. Like the first test, for each pattern we declare a program the "winner" if it has the highest performance scores. In order to take into account the rank of the pattern we compute the ratio of rank to average-site-score.

**Figure 6:**
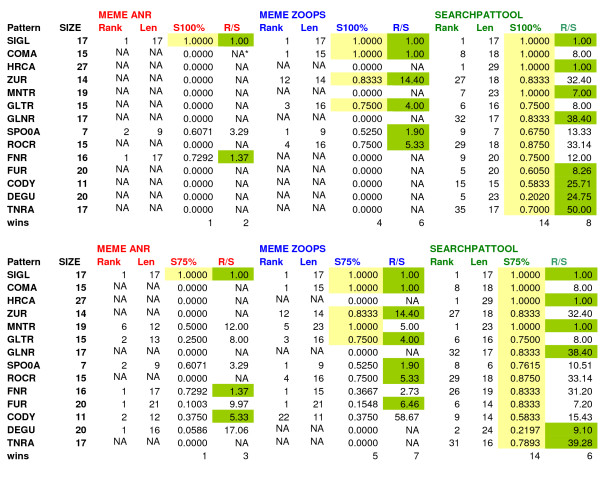
**Performance comparison of different programs for 100% and 75% minimum overlap**. Above, for each TF pattern, we indicate its correct sizes; and the rank, length (Len), average site scores for a minimum overlap of 100% (S100%), and ratio rank/S100% (R/S) for respectively Meme (anr and zoops) and Searchpattool. Below, for each TF pattern, we indicate its correct sizes; and the rank, length (Len), average site scores for a minimum overlap of 75% (S75%), and ratio rank/S75% (R/S) for respectively Meme (anr and zoops) and Searchpattool. Yellow and green background colours indicate respectively the best performance score and the best ratio. *Na = not available

Figure 6 shows the results for the best performance scores relative to each program for a minimum percentage of overlap equal to 100%. When comparing the performance site score (S100%), Searchpattool wins all the time and Meme (zoops) ties it four times. Searchpattool outperforms Meme (zoops) on ten TF. When comparing the ratio of rank to average-site-score (R/S), Searchpattool is also the leader (wins eight times) followed by Meme zoops (wins six times). We note that whenever Meme succeeds in retrieving a pattern and has a score different than zero then the ratio is always better than Searchpattool. In Meme the correct length is reported only for three TF SigL, Coma and Zur. Figure 6 shows also the results for a minimum percentage of overlap equal to 75%. When comparing the performance site score (S75%), Searchpattool is always the best. It has better scores for Spo0a, Fur and Tnra patterns. Their lengths are smaller than the known ones. Meme (Zoops) has more pattern candidates for other TF and has better ratio scores than Searchpattool (Meme wins seven times and Searchpattool wins six times).

**Figure 7:**
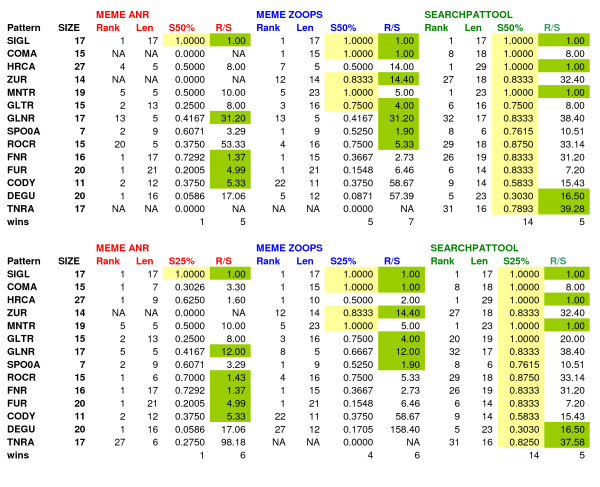
**Performance comparison of different programs for 50% and 25% minimum overlap**. Above, for each TF pattern, we indicate its correct sizes; and the rank, length (Len), average site scores for a minimum overlap of 50% (S75%), and ratio rank/S50% (R/S) for respectively Meme (anr and zoops) and Searchpattool. Below, for each TF pattern, we indicate its correct sizes; and the rank, length (Len), average site scores for a minimum overlap of 25% (S25%), and ratio rank/S25% (R/S) for respectively Meme (anr and zoops) and Searchpattool. Yellow and green background colours indicate respectively the best performance score and the best ratio. *Na = not available

Similar results are reported when the minimum rate of overlap is 50% or 25% (see Figure 7). The performance site scores are still the best for Searchpattool. Some patterns scores are improved, such as Degu (S50%), Gltr (S25%) and Tnra (S25%). Meme (zoops) has more candidates. Its performance site scores are not better than those of Searchpattool however, it has the best ratio scores.

### Searchpattool runtime and number of patterns study

**Figure 8:**
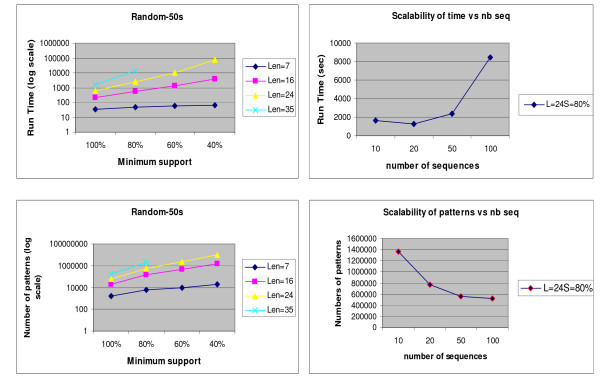
**Searchpattool runtime and number of patterns study**. At left, we run Searchpattool many times on a random input of 50 sequences by varying the value for the minimum support (from 100% to 40%) and the value for the maximum length of the pattern (from 7 to 35); and we record its running time and the total number of patterns. At right, we run Searchpattool on four random input sequences with respectively 10, 20, 50 and 100 sequences (400 bp for each sequence). For each input sequence we choose a minimum support value of 80% and a maximum length pattern value of 24, execute Searchpattool and record its running time and the total number of patterns.

We measured Searchpattool's runtime and its total number of patterns by varying the support and the maximum length of patterns. The runtime corresponds to the time for generating and scoring all frequent patterns. It does not include the running time for step 4. Since our input sequences are assumed to be derived from a small cluster of related genes, we generated a random set of 50 input sequences -each of length 400 bp- using the background probabilities of *Bacillus subtilis*. We chose four values for the support: 100%, 80%, 60% and 40%. We selected four values for the maximum length: 7, 16, 24 and 35. We note here that the runtime includes the calculation of the two z-scores, zs-sup and zs-tot. The performance of Searchpattool depends on several factors, including the number of input sequences, their lengths, and the user-specified values for the minimum support and the maximum length of target patterns. Figure 8 shows that as the value of the support decreases and the value of the length increases then the run time increases. The effect is similar for the total number of patterns. We ran Searchpattool on windows XP that allows only a maximum of 2 GB of memory per process. When the length was fixed to 35 and the minimum support was set to 60% or 40%, Searchpattool suffered from lack of memory and we were unable to report values for the run time and the number of patterns. We note that the performance of Searchpattool depends essentially on the user-specified maximum length of the patterns. As this quantity grows the time needed grows correspondingly.

In order to study the scalability of Searchpattool with different numbers of sequences we generated 3 sets of 10, 20 and 100 sequences, respectively. Each sequence has 400 bp. We then ran Searchpattool with a maximum length of 24 and a minimum support value of 80%. We chose these values because they are suitable for a study of a set of linked genes in *Bacillus subtilis*. Our experiments (see Figure 8) showed that as the number of sequences increased the runtime increased. Interestingly, however, the number of patterns decreased. In fact, Searchpattool spent more time searching common patterns of large number of sequences, which reduced the chance of finding a large number of common patterns. Detailed results can be found in the additional files [see Additional file 4].

## Discussion

The problem of discovering patterns for binding sites is complex and it is difficult to know a priori the kind of pattern to search for (organization, size or location). Searchpattool looks for patterns that contain at least 2 conserved bases with a conserved base at the beginning and the end. Its pattern model is derived from observations of several known patterns of transcription factors relative to prokaryotic species. Searchpattool is based on an exhaustive algorithm that searches for all frequent patterns of different sizes (from 2 to the specified-maximum length) with useful information like the support, density, length, zs-sup, zs-tot and list of positions. The user can view them and select the best ones according to personal criteria. We chose to select the best patterns initially according to the rank of the Z-score of the support (zs-sup). This score is very interesting for prokaryotic species where many motifs are rare, and it avoids the problem of overlapping motifs. For these best patterns we provide the user with additional information including their sites, matrices of frequencies, consensus, mean of information content and information about their reverse complements and mutual similarities.

Our scoring function allows extraction of the most specific patterns that are large and dense (the conservative patterns). These patterns will have the best zs-sup scores. By looking at the similarity matrix we can search for less specific similar ones. Finally we compute the p-value of zs-sup of selected patterns to check if these patterns are statistically significant. We propose to select the ones which have the best p-value and the highest support value. In cases where many patterns have the same rank (same p-value and same support) the user can refine the selection by looking at information about reverse complement, similarity and information content.

Comparing the accuracy of Searchpattool to that of well known local multiple alignment algorithms Meme, Motif sampler and Consensus, our experiments on 14 input sequences have shown that Searchpattool performs very well based on performance scores. In fact, Searchpattool does better than the other algorithms based on the performance score every time when the length is not restricted. However due to its algorithm type, the rank of the patterns are not always the best, especially when compared with Meme which is a local multiple alignment algorithm. In order to take into account the rank, we calculated the ratio of rank to performance-score which measures the precision of each algorithm. Our study shows that Meme performs the best based on these ratios when the pattern length is fixed. If the length is not restricted, Searchpattool has the best rate when the overlap percentage is set to 100%. Searchpattool performs very well for sets of very small numbers of sequences (2 or 3), but Meme fails to retrieve the patterns for those cases. Comparing Searchpattool to the exhaustive algorithm Mitra, our experiments have shown that Searchpattool is more accurate and has better ranking. Searchpattool also can search larger patterns.

Our study shows that Searchpattool performs very well and outputs the most specific patterns that correspond to the known motifs or closer similar ones. However, there were some TF (like Spo0A, Fur or Tnra) where Meme, Consensus or Mitra did better. When the known motif conformed to its model, Searchpattool performed better, as expected. However, there are cases where the known motif cannot be reasonably captured by Searchpattool's motif model. Hence, we suggest using some other motif finding algorithms in conjunction to Searchpattool, for better accuracy.

Many factors affect the runtime and the output of Searchpattool, including the number of input sequences, the values for the minimum support and the maximum length of the target patterns. As the number of sequences increases the run time increases as well. In addition, Searchpattool outputs more patterns and consumes more time when the length is greater than 24 and the support is less than 80%. We note that for our testing we generated random samples with lengths fixed at 400 bp (the maximum upstream length for prokaryotic species) but we know from well-known regulons that the upstream region can be as small as a dozen base pairs, so in practice we may often have smaller runtimes. On the other hand, if we use input sequences from regulon sets instead of random sets then the genes are more correlated and we may get a larger number of (frequent) patterns. For instance Searchpattool finds 46978 patterns for SigL TF (with min-sup = 6 and max-len = 17), whereas the corresponding maximum number of patterns found from the 1000 random samples is 40020.

For the future we are working on improving the precision of Searchpattool. We are thinking about extending it to allow some ambiguous characters in order to have more general patterns. We also plan to test Searchpattool on other species, and to implement higher orders of Hidden Markov Models.

In order to reduce the runtime we will work on a parallel version of Searchpattool. In fact we can easily process each family of pattern separately and this will improve the performance. We will continue improving Searchpattool's interface and integration of the other programs. We will develop a web application with the same interface for all tools.

## Conclusion

We have presented a new method, Searchpattool, for TFBS pattern discovery based on an exhaustive algorithm. Searchpattool looks for the most specific or conservative patterns shared by a set of sequences. Our testing on *Bacillus subtilis *datasets shows that it performs very well and is efficient for small numbers of sequences. It is easy to use and provides rich and complete information about the best patterns. Either alone or as a complement to other algorithms, Searchpattool can be a powerful tool for discovering novel and important TFBS patterns common to a cluster of genes.

## Methods

Algorithm Searchpattool (input_sequence, min_support, max_length, background-proba, #output)

Begin

   - Read the n input sequence and make their reverse complement.

   - Create the 4 arrays Tab_i _that contains the positions of corresponding nucleotide in the 2n sequences.

   - **For **i = 1 to 4   //each i references a nucleotide N_i_

         ○ For j = 1 to 4   **//step 1**

               ■ Join (Tab_i_, Tab_j_, LFP_ij_, N_i_, N_j_, min_support, max_length)

               ■ //create a LFP_ij _that start with N_i _and finish with N_j_.

            End for

         ○ Merge and sort by length all LFP_ij _to LFP_i_.

         ○ More-specific(LFP_i_, min_support)   **//step 2**

         ○ Statistics(LFP_i_, Results_i_)   **//step 3**

      **End for**

   - Sort-Extract-info(All_results, #output)   **//step 4**

End

Searchpattool begins by reading the n input sequence and making their reverse complement. Then it allocates 4 arrays (of 2n elements) relative to the 4 nucleotides. Each array (Tab_i_) contains the positions of corresponding nucleotide in the 2n sequences (in the double strands).

The module 'Join' creates the lists of patterns of the form E (.)* E. For each nucleotide i it creates the list of all frequent patterns (LFP_i_) that start with i and finish with all the 4 others with just wild-characters in the middle The number of wild-character varies from 0 to max-length-2. The module 'More-specific' makes a list of patterns of the form E (.)* E more specific by replacing wild-character with nucleotides. The format of the pattern will be E (E ∪ .)* E. The module 'Statistics' computes the Z-scores of all patterns by family and store the results in text files. Finally the module 'Sort-Extract-info' sorts the patterns according to the z-score of the support and selected the desired number of patterns with all useful information.

### Computation of the z-scores

For each pattern P we define these variables:

p = probability of P at any given position

n = number of sequences in the sequence set.

L_j _= length of the j^th ^sequence

k = length of P

s = support of P over the n sequences

o = number of observed matching positions of P without overlapping positions

T = the number of possible matching positions of P

T = (2 * Σ_j = 1, n _(L_j _+ 1 - k)) - o * (k-1)

The count of T does not allow overlapping positions in the double strands. For each occurrence of P we exclude the next k-1 positions from the count.

The expected occurrences of P (eo) is:

eo = p * T

The variance (vo)is:

vo = T * p * (1-p).

The z-score for total number of occurrences of pattern P is:

Zs-tot = (o-eo)/sqrt(vo).

The probability that there will be at least one occurrence of pattern P within the sequence 'j' (q_j_) is [24]:

q_j _= 1 - (1-p) T_j _where T_j _= 2 * (L_j_-k+1).

The expected number of matching sequences (es) is:

es = Σ_j = 1,n _(q_j_).

We can estimate the variance of the support (vs) under an independence assumption as

vs = Σ_j = 1,n _((1- q_j_) q_j_)

The z-score of the support of pattern P is:

zs-sup = (s-es)/sqrt(vs).

For this statistic, we do not assume it will have a Gaussian distribution, nor do we need the independence assumption to hold, since the significance of the score is computed via simulations from random data. For our experiments we assumed that the four nucleotides are independent so the background probability p of pattern P at a position is the product of the background probabilities of well conserved nucleotides contained in P.

### Scoring by information content

We can score selected patterns according to their information content. Given a pattern of length k and its list of instances, the information content (IC) of this pattern is defined to be

IC = Σ_j = 1,k_Σ_c _p_c,j _log_2 _p_c,j_/b_c_

Where p_c,j _is the frequency with which character c (A,G,C or T) occurs in position j among the pattern occurrences and b_c _is the background frequency of c.

Since we have patterns of different lengths we take the mean (average) of the information content (MIC) as a second score for significance

MIC = [Σ_j = 1,k_Σ_c _p_c,j _log_2 _p_c,j_/b_c_]/k

The MIC score is useful for refining the selection of the best patterns.

### Computing the p-values of the best z-scores

For each experiment, we select the 'h' highest scored motifs zs-sup_i _(with 1<=i<=h) and for each zs-sup_i _we compute a probability p-value_i _that the i^th ^maximum score chosen by our selection process would be at least zs-sup_i _if the input sequences were random. To calculate this probability in an unbiased way we use the following approach: first, we generate random samples of the same length and in the same quantity as the input sequences using the background probabilities of the four nucleotides. For each random sample we run Searchpattool with the same parameters as the original experiment and select the best 'h' zs-sup* scores; denote these zs-sup_1_*, zs-sup_2_*, ... zs-sup_h_*, ordered from largest to smallest. We repeat this process for 1000 different sets of randomly generated sequences. The end result is that we have a distribution of 1000 of each of the maximum zs-sup_1_*, the second-to-highest zs-sup_2_*, and so forth to the h^th^-highest. Each of our observed zs-sup_i _is then compared to the distribution of the equivalent order statistics from the randomly generated samples. We compute the p-value corresponding to the zs-sup_i _as the proportion of zs-sup_i_*>= zs-sup_i_. This allows us to account for the selection process in computing the probability that a particular motif would have support as high as was observed by chance. Note that based on these 1000 randomly generated sequence sets the lowest possible p-value for any motif is <0.001, corresponding to the case when none of the zs-sup_i_* are greater or equal to the observed zs-sup_i_.

### Nucleotide level performance score

For each pattern we define TP,FN,FP as respectively the number of nucleotides positions in both known sites and predicted sites, the number of nucleotide positions in known sites but not in predicted sites and the number of nucleotide positions not in known sites but in predicted sites. The nucleotide level performance score (ps) is defined as TP/(TP+FN+FP).

### Average site level performance score

For each pattern we define STP, SFN, SFP as respectively the number of known sites overlapped by predicted sites, the number of known sites not overlapped by predicted sites, and the number of predicted sites not overlapped by known sites. The site sensitivity score is defined as STP/(STP+SFN). The site positive predictive value score is defined as STP/(STP+SFP). The average site score is the average of the sensitivity score and positive predictive scores. We compute this score for a minimum overlap percentage taking respectively the values 100 (S100%), 75 (S75%), 50 (S50%) and 25 (S25%).

## Availability and requirements

Searchpattool software is available as an additional file with the article [see Additional file 5]. It is hosted at bioinformatics.org and is also accessible via ftp: 

**Operating system: **Windows

**Programming language: **C++

**Other requirements: **none

**License: **GNU GPL

**Any restrictions to use by non-academics: **none

## Abbreviations

TF, transcription factor; TFBS, transcription factor binding sites

## Competing interests

The author(s) declares that there are no competing interests.

## Authors' contributions

**FE **designed the algorithm, developed the software tool, conducted the whole study and drafted the manuscript. **MN **participated in the statistical study, proposed the formula of the z-score of the support and the method to compute the p-value of the z-scores, wrote the R script for generating random samples and revised the manuscript. All authors read and approved the final manuscript.

## Supplementary Material

Additional file 1 The data sets. This file contains the input sequences and known sites for each TF used in our experiments. Click here for file

Additional file 2 Searchpattool's results for test1. This file contains the best patterns as predicted by Searchpattool for each of the fourteen TF examined in test1. Click here for file

Additional file 3 Searchpattool's results for test2. This file contains the best patterns as predicted by Searchpattool for each of the fourteen TF examined in test2. Click here for file

Additional file 4 Runtime and number of patterns statistics. This file contains detailed results for the study of runtime and number of patterns found by Searchpattool on random data sets. Click here for file

Additional file 5 Searchpattool's software. This archive file contains the application, the source code; a runable example and an explanation of the use of the programs (file readme-searchpattool-doc.pdf) Click here for file
