# Supplementary material for: SEARCHPATTOOL: a new method for mining the most specific frequent patterns for binding sites with application to prokaryotic DNA sequences
Source: BMC Bioinformatics. 2007 Sep 20;8:354. doi: 10.1186/1471-2105-8-354 (PMC2082047; doi:10.1186/1471-2105-8-354)
Supplement: Additional file 5 — Searchpattool's software. This archive file contains the application, the source code; a runable example and an explanation of the use of the programs (file readme-searchpattool-doc.pdf) [file 1471-2105-8-354-S5.zip › example&doc/readme-searchpattool-doc.pdf]

SEARCHPATTOOL is a program for analyzing DNA sequences in order to discover patterns or motifs for binding sites. It is dedicated to prokaryotic species or genes with small promoter regions. Given a set V of N sequences, a subset C (of V) of size n, and a pattern P that occurs in‘s’ sequences from C and matches ‘o’ positions in C (with counting the double strands), we can ask first how probable is the event of observing the pattern P to match s or more sequences of C and second how probable is the event of observing the pattern P to match o or more positions in C. We compute the z-score of the number of sequences or support (zs-sup) and the z-score of the total of positions (zs-tot). The selected patterns are those of the best z-scores. Other associated programs are developed that help the user to choose the best motifs based on statistical assessments.
All programs are developed and tested under Windows XP operating system. They are Dos application Windows. If you are using a Windows system and my programs do not work, please notify me.

CONTENTS

--Main Programs ---

1- SEARCHPATTOOL: outputs E (E U ‘.’)* E formatted patterns where E represent a nucleotide (A,C,G,T), the character ‘.’ is the wild-character N, U represents the operator union and * the repetition from 0 to n times. For each pattern it reports its length, density, support, z-scores and list of occurrences. We choose to limit the search for maximum of 40bp patterns. An input sequence should be in Fasta format (now up to 500bp).

<u>Call:</u>
C>**searchpattool** <*input-file-name*> **minsupport** <*minimum-support-value*> **maxlength** <*maximum-pattern-length*> [**stats** <*pA*> <*pC*> <*pG*> <*pT*>] [**output** <*number-of-patterns*>]

<u>Parameters:</u>

input-file-name:	it is the input sequences in Fasta format (text file)
minimum-support-value:	it the minimum number of sequences that contains the motif (minimum 2)
maximum-pattern-length:	Searchpatt outputs patterns of different length from 2 to this specified value (maximum 40)
pA, pC, pG, pT:	background probabilities for A, C, G and T of studied species. Bacillus Subtilis values are set by default.
number-of-patterns:	number of patterns to output (now: default 40, max 2000)

<u>Output:</u>
Main results

- **bestzssup.txt**: contains the list of patterns ordered by their z-score of the support with information including their length, density, support, z-scores and list of occurrences.
- **Sites-positions.txt**: contains the sites and the exact positions of the patterns
- **Sites-for-logos.txt**: contains just the sites in order to display logos
- **Patt-profiles.txt**: contains the frequency matrices of the patterns

- **Patt-cons-mic.txt**: contains the list of pattern plus their consensus and their mean information content
- **Patt-rev-com.txt**: indicates for each pattern its reverse complement on the list of pattern
- **Patt-similarity.txt**: measures the degree of similarity between the listed patterns
- **Runreport.txt**: reports information about the current results after Searchpattool and other programs runs.

Internal files (don't erase them!)
- resA.txt: all patterns that begin with A
- resC.txt: all patterns that begin with C
- resG.txt: all patterns that begin with G
- resT.txt: all patterns that begin with T

Other files: finfo.txt, frev.txt, ftot.txt and fproba.txt

2- SEARCH_BEST_RANDOM_SCORES: a program that computes the best z-scores of the support (zs-sup) relative to 1000 random samples (an R script is provided to generate random samples). These samples should be provided in the same directory of this program ('sample1.txt', 'sample2.txt', … , 'sample1000.txt'). The user should provide the same parameters used in Searchpattool call including the number of outputs.

<u>Call:</u>
C>**search_best_random_scores minsupport** <*minimum-support-value*> **maxlength** <*maximum-pattern-length*> [**stats** <*pA*> <*pC*> <*pG*> <*pT*>] [**output** <*number-of-patterns*>]

<u>Parameters:</u>

minimum-support-value:	it the minimum number of sequences that contains the motif (minimum 2)
maximum-pattern-length:	Searchpatt outputs patterns of different length from 2 to this specified value (maximum 40)
pA, pC, pG, pT:	background probabilities for A, C, G and T of studied species. Bacillus Subtilis values are set by default.
number-of-patterns:	number of patterns to output

<u>Output:</u>
- **Random-scores.txt**: contains the list of the best z-scores of the support for the 1000 random samples.
- Internal files

<u>Remark:</u> It is better to run this program in a directory different of that Searchpattool

3- COMPUTE_PVALUE: a program that computes the p-value of the best zs-sup relative to the current patterns on our list by using the random scores. The files 'random-scores.txt', 'patt-cons-mic.txt' and 'patt-rev-com.txt' are necessary for the execution of this program.

Call:
C>**compute_pvalue**

Output:
- **Final-results-with-pvalue.txt**: contains the list of best patterns with all statistics.

The figure below gives an overview of the order of use of the main programs.

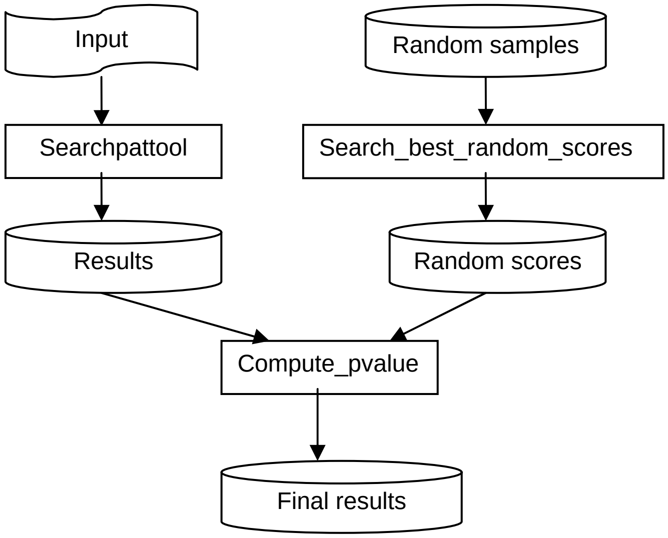

-- Utilities --

4- SELECT-BEST-ZS-SUP: a program that outputs the n best patterns ordered by their z-score of the support. A run of Searchpattool should precede it. This program is useful if the user wants to specify a number of outputs different from that used in the call of Searchpattool. It avoids searching again for all patterns with the same parameters.

Call:
C>**select-best-zs-sup** [<*number-of-patterns*>]

Parameters:
number-of-patterns: number of patterns to output (now: default 40, min 10, max 2000)

Output:
- **bestzssup.txt**: contains the list of patterns ordered by their z-score of the support with information including their length, density, support, z-scores and list of occurrences.
- **Sites-positions.txt**: contains the sites and the exact positions of the patterns
- **Sites-for-logos.txt**: contains just the sites in order to display logos
- **Patt-profiles.txt**: contains the frequency matrices of the patterns
- **Patt-cons-mic.txt**: contains the list of pattern plus their consensus and their mean information content
- **Patt-rev-com.txt**: indicates for each pattern its reverse complement on the list of pattern

- **Patt-similarity.txt**: measures the degree of similarity between the listed patterns
- **Runreport.txt**: reports information about the current results (updated).

5- SELECT-BEST-ZS-TOT: is a program that outputs the best patterns ordered by their z-score of the total number of occurrences. A run of Searchpattool should precede it.

Call:
C>**select-best-zs-tot** [<*number-of-patterns*>]

Parameters:
Number-of-patterns: number of patterns to output (now: default 40, min 10, max 2000)

Output:
- **bestzstot.txt**: contains the list of patterns ordered by their z-score of the total number of occurrences with information including their length, density, support, z-scores and list of occurrences.
- **Sites-positions-tot.txt**: contains the sites and the exact positions of the patterns
- **Sites-for-logo-tot s.txt**: contains just the sites in order to display logos
- **Patt-profiles-tot.txt**: contains the frequency matrices of the patterns
- **Patt-cons-mic-tot.txt**: contains the list of pattern plus their consensus and their mean information content
- **Patt-rev-com-tot.txt**: indicates for each pattern its reverse complement on the list of pattern
- **Patt-similarity-tot.txt**: measures the degree of similarity between the listed patterns
- **Runreport.txt**: reports information about the current results (updated).

-- Example ---
We run searchpattool on Spo0A input sequences (promospo0abs.txt). Due to space limitations the 1000 random samples are not given but we use them to generate the random scores file 'random-scores.txt'. The R script is provided (see file Rscript.txt)

-- Remarks ---
-- Results format reading
All results are presented as text files but most of them can be read by MS Excel or JMP (for more than 256 columns)
-- Patterns similarity interpretation
The file **Patt-similarity.txt** measures the degree of similarity between the selected patterns. In fact for each pattern P (each pattern column in the file: patt1, patt2…) we check if it covers or extends (overlap 100%) another pattern Q (a row) and measure their degree of similarity by computing the average site similarity score (see paper). A similarity score of 1 means that P covers totally Q. So P is an extension of Q. A zero score means that P does not cover Q. A score between 0 and 1 means, that some sites of P cover some of Q.

Fathi Elloumi
felloumi@bioinformatics.org
